# Supplementary material for: Alkaline water as a potential agent for biting midge control: Managing effectiveness and non-target organism impact evaluation
Source: PLoS One. 2023 Aug 17;18(8):e0290262. doi: 10.1371/journal.pone.0290262 (PMC10434854; doi:10.1371/journal.pone.0290262)
Supplement: S1 File — (DOCX) [file pone.0290262.s001.docx]

S1 Fig.1. Table of the eggs number of *Forcipomyia taiwana* (after transformation)

| Data | Treatment | num_sqr | num_log |
| --- | --- | --- | --- |
| 5C | C | 11.61895004 | 2.133538908 |
| 2C | C | 5.385164807 | 1.477121255 |
| 3C | C | 14.62873884 | 2.33243846 |
| 4C | C | 11.26942767 | 2.10720997 |
| 6C | C | 9.055385138 | 1.919078092 |
| 7C | C | 4.582575695 | 1.342422681 |
| 8C | C | 11 | 2.086359831 |
| 9C | C | 9.486832981 | 1.959041392 |
| 10C | C | 11 | 2.086359831 |
| 11C | C | 12.52996409 | 2.198657087 |
| 12C | C | 5.291502622 | 1.462397998 |
| 13C | C | 8.602325267 | 1.875061263 |
| 15C | C | 4.69041576 | 1.361727836 |
| 16C | C | 7.211102551 | 1.72427587 |
| 17C | C | 7.483314774 | 1.755874856 |
| 5T | T | 0 | 0 |
| 2T | T | 9.219544457 | 1.934498451 |
| 3T | T | 6.92820323 | 1.69019608 |
| 4T | T | 7.745966692 | 1.785329835 |
| 6T | T | 9.273618495 | 1.939519253 |
| 7T | T | 5.567764363 | 1.505149978 |
| 8T | T | 10.44030651 | 2.041392685 |
| 9T | T | 3.605551275 | 1.146128036 |
| 10T | T | 6.782329983 | 1.672097858 |
| 11T | T | 8.94427191 | 1.908485019 |
| 12T | T | 6.164414003 | 1.591064607 |
| 13T | T | 10.29563014 | 2.029383778 |
| 15T | T | 7 | 1.698970004 |
| 16T | T | 5 | 1.414973348 |
| 17T | T | 7.141428429 | 1.716003344 |

S2 Fig.2. Table of the eggs number of cricket

| Data | Number | Treatment | Data | Number | Treatment |
| --- | --- | --- | --- | --- | --- |
| C1 | 315 | C | T1 | 121 | T |
| C2 | 205 | C | T2 | 159 | T |
| C3 | 27 | C | T3 | 98 | T |
| C4 | 82 | C | T4 | 35 | T |
| C5 | 0 | C | T5 | 0 | T |
| C7 | 78 | C | T7 | 29 | T |
| C8 | 161 | C | T8 | 32 | T |
| C9 | 36 | C | T9 | 116 | T |
| C10 | 166 | C | T10 | 208 | T |
| C11 | 8 | C | T11 | 58 | T |
| C12 | 4 | C | T12 | 28 | T |
| C14 | 15 | C | T14 | 3 | T |
| C15 | 0 | C | T15 | 1 | T |
| C16 | 65 | C | T16 | 12 | T |
| C17 | 139 | C | T17 | 116 | T |
| C18 | 51 | C | T18 | 444 | T |
| C19 | 220 | C | T19 | 272 | T |
| C20 | 0 | C | T20 | 2 | T |
| C21 | 64 | C | T21 | 7 | T |
| C22 | 0 | C | T22 | 0 | T |
| C23 | 12 | C | T23 | 103 | T |

S3 Fig.3. Table of Earthworm distribution

| Data | Tray | Earthworm number | Treatment | Segment | Data | Tray | Earthworm number | Treatment | Segment |
| --- | --- | --- | --- | --- | --- | --- | --- | --- | --- |
| T1_1 | 1 | 16 | T | T1 | C1_1 | 1 | 12 | C | C1 |
| T1_2 | 2 | 20 | T | T1 | C1_2 | 2 | 20 | C | C1 |
| T1_3 | 3 | 14 | T | T1 | C1_3 | 3 | 19 | C | C1 |
| T1_4 | 4 | 26 | T | T1 | C1_4 | 4 | 31 | C | C1 |
| T1_5 | 5 | 29 | T | T1 | C1_5 | 5 | 19 | C | C1 |
| T1_6 | 6 | 35 | T | T1 | C1_6 | 6 | 32 | C | C1 |
| T1_7 | 7 | 24 | T | T1 | C1_7 | 7 | 19 | C | C1 |
| T1_8 | 8 | 20 | T | T1 | C1_8 | 8 | 25 | C | C1 |
| T1_9 | 9 | 37 | T | T1 | C1_9 | 9 | 35 | C | C1 |
| T1_10 | 10 | 19 | T | T1 | C1_10 | 10 | 22 | C | C1 |
| T1_11 | 11 | 55 | T | T1 | C1_11 | 11 | 73 | C | C1 |
| T1_12 | 12 | 44 | T | T1 | C1_12 | 12 | 42 | C | C1 |
| T1_13 | 13 | 57 | T | T1 | C1_13 | 13 | 44 | C | C1 |
| T1_14 | 14 | 32 | T | T1 | C1_14 | 14 | 29 | C | C1 |
| T1_15 | 15 | 34 | T | T1 | C1_15 | 15 | 51 | C | C1 |
| T1_16 | 16 | 36 | T | T1 | C1_16 | 16 | 30 | C | C1 |
| T2_1 | 1 | 13 | T | T2 | C2_1 | 1 | 16 | C | C2 |
| T2_2 | 2 | 22 | T | T2 | C2_2 | 2 | 23 | C | C2 |
| T2_3 | 3 | 23 | T | T2 | C2_3 | 3 | 29 | C | C2 |
| T2_4 | 4 | 28 | T | T2 | C2_4 | 4 | 28 | C | C2 |
| T2_5 | 5 | 29 | T | T2 | C2_5 | 5 | 18 | C | C2 |
| T2_6 | 6 | 39 | T | T2 | C2_6 | 6 | 29 | C | C2 |
| T2_7 | 7 | 39 | T | T2 | C2_7 | 7 | 28 | C | C2 |
| T2_8 | 8 | 17 | T | T2 | C2_8 | 8 | 18 | C | C2 |
| T2_9 | 9 | 26 | T | T2 | C2_9 | 9 | 47 | C | C2 |
| T2_10 | 10 | 34 | T | T2 | C2_10 | 10 | 36 | C | C2 |
| T2_11 | 11 | 28 | T | T2 | C2_11 | 11 | 25 | C | C2 |
| T2_12 | 12 | 34 | T | T2 | C2_12 | 12 | 58 | C | C2 |
| T2_13 | 13 | 42 | T | T2 | C2_13 | 13 | 49 | C | C2 |
| T2_14 | 14 | 26 | T | T2 | C2_14 | 14 | 29 | C | C2 |
| T2_15 | 15 | 50 | T | T2 | C2_15 | 15 | 46 | C | C2 |
| T2_16 | 16 | 54 | T | T2 | C2_16 | 16 | 33 | C | C2 |

S4 Fig.4. Table of biting midge *Forcipomyia taiwana* density in the field

| **Plot** | **Number** | **Time** | **Plot 2** | **Trt** | **Trt2** | **Plot** | **Number** | **Time** | **Plot 2** | **Trt** | **Trt2** |
| --- | --- | --- | --- | --- | --- | --- | --- | --- | --- | --- | --- |
| bP1_1 | 0.477121255 | before | P1_1 | T | bT | dP17_2 | 0.84509804 | during | P17_2 | C | dC |
| bP1_2 | 0.477121255 | before | P1_2 | C | bC | dP2_2 | 0.84509804 | during | P2_2 | C | dC |
| bP11_1 | 0.77815125 | before | P11_1 | T | bT | dP5_2 | 0.84509804 | during | P5_2 | C | dC |
| bP11_2 | 0.77815125 | before | P11_2 | C | bC | dP7_1 | 0.84509804 | during | P7_1 | T | dT |
| bP12_1 | 1.041392685 | before | P12_1 | T | bT | dP12_2 | 0.77815125 | during | P12_2 | C | dC |
| bP12_2 | 1.204119983 | before | P12_2 | C | bC | dP13_1 | 0.77815125 | during | P13_1 | T | dT |
| bP13_1 | 1.431363764 | before | P13_1 | T | bT | aP13_2 | 0.77815125 | after | P13_2 | C | aC |
| bP13_2 | 1.431363764 | before | P13_2 | C | bC | dP17_1 | 0.698970004 | during | P17_1 | T | dT |
| bP14_1 | 1.322219295 | before | P14_1 | T | bT | dP2_1 | 0.698970004 | during | P2_1 | T | dT |
| bP14_2 | 1.278753601 | before | P14_2 | C | bC | dP6_2 | 0.698970004 | during | P6_2 | C | dC |
| bP15_1 | 1.431363764 | before | P15_1 | T | bT | aP20_2 | 0.698970004 | after | P20_2 | C | aC |
| bP15_2 | 1.322219295 | before | P15_2 | C | bC | dP1_1 | 0.602059991 | during | P1_1 | T | dT |
| bP17_1 | 1 | before | P17_1 | T | bT | dP1_2 | 0.602059991 | during | P1_2 | C | dC |
| bP17_2 | 1.176091259 | before | P17_2 | C | bC | dP8_1 | 0.602059991 | during | P8_1 | T | dT |
| bP18_1 | 0.477121255 | before | P18_1 | T | bT | aP12_2 | 0.602059991 | after | P12_2 | C | aC |
| bP18_2 | 0.903089987 | before | P18_2 | C | bC | aP20_1 | 0.602059991 | after | P20_1 | T | aT |
| bP2_1 | 0.77815125 | before | P2_1 | T | bT | aP7_1 | 0.602059991 | after | P7_1 | T | aT |
| bP2_2 | 1.041392685 | before | P2_2 | C | bC | dP12_1 | 0.477121255 | during | P12_1 | T | dT |
| bP20_1 | 1.491361694 | before | P20_1 | T | bT | dP5_1 | 0.477121255 | during | P5_1 | T | dT |
| bP20_2 | 1.556302501 | before | P20_2 | C | bC | aP11_2 | 0.477121255 | after | P11_2 | C | aC |
| bP5_1 | 0.903089987 | before | P5_1 | T | bT | aP13_1 | 0.477121255 | after | P13_1 | T | aT |
| bP5_2 | 1 | before | P5_2 | C | bC | aP17_2 | 0.477121255 | after | P17_2 | C | aC |
| bP6_1 | 0.698970004 | before | P6_1 | T | bT | aP5_1 | 0.477121255 | after | P5_1 | T | aT |
| bP6_2 | 0.84509804 | before | P6_2 | C | bC | aP8_2 | 0.477121255 | after | P8_2 | C | aC |
| bP7_1 | 0.77815125 | before | P7_1 | T | bT | dP6_1 | 0.301029996 | during | P6_1 | T | dT |
| bP7_2 | 0.477121255 | before | P7_2 | C | bC | dP7_2 | 0.301029996 | during | P7_2 | C | dC |
| bP8_1 | 0.954242509 | before | P8_1 | T | bT | dP8_2 | 0.301029996 | during | P8_2 | C | dC |
| bP8_2 | 0.698970004 | before | P8_2 | C | bC | aP1_1 | 0.301029996 | after | P1_1 | T | aT |
| bP9_1 | 1.278753601 | before | P9_1 | T | bT | aP1_2 | 0.301029996 | after | P1_2 | C | aC |
| bP9_2 | 1.322219295 | before | P9_2 | C | bC | aP11_1 | 0.301029996 | after | P11_1 | T | aT |
| dP14_2 | 1.491361694 | during | P14_2 | C | dC | aP17_1 | 0.301029996 | after | P17_1 | T | aT |
| aP14_2 | 1.431363764 | after | P14_2 | C | aC | aP18_2 | 0.301029996 | after | P18_2 | C | aC |
| dP20_2 | 1.278753601 | during | P20_2 | C | dC | aP2_1 | 0.301029996 | after | P2_1 | T | aT |
| aP15_2 | 1.230448921 | after | P15_2 | C | aC | aP6_2 | 0.301029996 | after | P6_2 | C | aC |
| dP14_1 | 1.204119983 | during | P14_1 | T | dT | aP8_1 | 0.301029996 | after | P8_1 | T | aT |
| dP20_1 | 1.204119983 | during | P20_1 | T | dT | aP9_1 | 0.301029996 | after | P9_1 | T | aT |
| aP14_1 | 1.204119983 | after | P14_1 | T | aT | aP9_2 | 0.301029996 | after | P9_2 | C | aC |
| dP13_2 | 1.079181246 | during | P13_2 | C | dC | dP18_1 | 0 | during | P18_1 | T | dT |
| dP15_1 | 1.079181246 | during | P15_1 | T | dT | dP18_2 | 0 | during | P18_2 | C | dC |
| dP9_1 | 1.079181246 | during | P9_1 | T | dT | aP12_1 | 0 | after | P12_1 | T | aT |
| dP11_1 | 1.041392685 | during | P11_1 | T | dT | aP18_1 | 0 | after | P18_1 | T | aT |
| dP15_2 | 1.041392685 | during | P15_2 | C | dC | aP2_2 | 0 | after | P2_2 | C | aC |
| dP9_2 | 1 | during | P9_2 | C | dC | aP5_2 | 0 | after | P5_2 | C | aC |
| dP11_2 | 0.954242509 | during | P11_2 | C | dC | aP6_1 | 0 | after | P6_1 | T | aT |
| aP15_1 | 0.903089987 | after | P15_1 | T | aT | aP7_2 | 0 | after | P7_2 | C | aC |
|  |  |  |  |  |  |  |  |  |  |  |  |

| Plot | Treatment | Chlorophyll_log(n+1) | Chlorophyll_sqrt |
| --- | --- | --- | --- |
| C1 | C | 0.07114529 | 0.421900462 |
| C2 | C | 0.132080298 | 0.59618789 |
| C5 | C | 0.18558748 | 0.73017806 |
| C6 | C | 0.41488648 | 1.264705499 |
| C7 | C | 0.255209769 | 0.894281835 |
| C8 | C | 0.059979717 | 0.384837628 |
| C9 | C | 0.07104205 | 0.4215685 |
| C11 | C | 0.41488648 | 1.264705499 |
| C12 | C | 0.236962782 | 0.851874404 |
| C13 | C | 0.159657411 | 0.666558325 |
| C14 | C | 0.125706758 | 0.579390484 |
| C15 | C | 0.150658214 | 0.64395652 |
| C17 | C | 0.25282068 | 0.888744433 |
| C18 | C | 0.221792288 | 0.816363889 |
| T1 | T | 0.070407322 | 0.419523539 |
| T2 | T | 0.087124512 | 0.471327911 |
| T5 | T | 0.15667835 | 0.659110512 |
| T6 | T | 0.327473369 | 1.060924125 |
| T7 | T | 0.201943063 | 0.769415362 |
| T8 | T | 0.063708559 | 0.397492138 |
| T9 | T | 0.117606004 | 0.557682705 |
| T11 | T | 0.117606004 | 0.557682705 |
| T12 | T | 0.18558748 | 0.73017806 |
| T13 | T | 0.087124512 | 0.471327911 |
| T14 | T | 0.136799811 | 0.608481717 |
| T15 | T | 0.081829589 | 0.455346022 |
| T17 | T | 0.127308934 | 0.58363516 |
| T18 | T | 0.276139985 | 0.942655823 |
|  |  |  |  |

S5 Fig.5. Table of the chlorophyll concentration (after transformation)

S6 Fig.6. Table of the abundance and morphospecies of ground insect and spider

| Plot | Time survey | Treatment | Insect abundance | Insect morfospecies | Spider abundance | Spider Morfospecies |
| --- | --- | --- | --- | --- | --- | --- |
| P1_1 | after | T | 2 | 2 | 0 | 0 |
| P1_2 | after | C | 14 | 11 | 2 | 2 |
| P11_1 | after | T | 12 | 9 | 0 | 0 |
| P11_2 | after | C | 9 | 7 | 0 | 0 |
| P12_1 | after | T | 84 | 16 | 3 | 3 |
| P12_2 | after | C | 8 | 7 | 0 | 0 |
| P13_1 | after | T | 93 | 15 | 2 | 2 |
| P13_2 | after | C | 1 | 1 | 0 | 0 |
| P14_1 | after | T | 64 | 17 | 1 | 1 |
| P14_2 | after | C | 326 | 18 | 0 | 0 |
| P15_1 | after | T | 146 | 5 | 0 | 0 |
| P15_2 | after | C | 11 | 8 | 0 | 0 |
| P17_1 | after | T | 18 | 13 | 1 | 1 |
| P17_2 | after | C | 50 | 21 | 2 | 2 |
| P18_1 | after | T | 7 | 3 | 0 | 0 |
| P18_2 | after | C | 552 | 20 | 2 | 2 |
| P2_1 | after | T | 10 | 8 | 0 | 0 |
| P2_2 | after | C | 18 | 11 | 0 | 0 |
| P20_1 | after | T | 6 | 5 | 1 | 1 |
| P20_2 | after | C | 15 | 7 | 0 | 0 |
| P5_1 | after | T | 60 | 5 | 1 | 1 |
| P5_2 | after | C | 5 | 3 | 0 | 0 |
| P6_1 | after | T | 30 | 8 | 0 | 0 |
| P6_2 | after | C | 76 | 8 | 0 | 0 |
| P7_1 | after | T | 5 | 5 | 0 | 0 |
| P7_2 | after | C | 12 | 9 | 0 | 0 |
| P8_1 | after | T | 9 | 7 | 1 | 1 |
| P8_2 | after | C | 12 | 5 | 0 | 0 |
| P9_1 | after | T | 19 | 6 | 0 | 0 |
| P9_2 | after | C | 70 | 7 | 0 | 0 |
| P1_1 | before | T | 23 | 14 | 6 | 1 |
| P1_2 | before | C | 42 | 22 | 3 | 3 |
| P11_1 | before | T | 24 | 10 | 17 | 3 |
| P11_2 | before | C | 15 | 11 | 4 | 2 |
| P12_1 | before | T | 11 | 8 | 1 | 1 |
| P12_2 | before | C | 9 | 7 | 4 | 4 |
| P13_1 | before | T | 33 | 7 | 3 | 3 |
| P13_2 | before | C | 20 | 7 | 2 | 2 |
| P14_1 | before | T | 16 | 12 | 8 | 3 |
| P14_2 | before | C | 45 | 18 | 1 | 1 |
| P15_1 | before | T | 3 | 2 | 0 | 0 |
| P15_2 | before | C | 11 | 5 | 4 | 3 |
| P17_1 | before | T | 41 | 19 | 4 | 3 |
| P17_2 | before | C | 24 | 17 | 8 | 4 |
| P18_1 | before | T | 47 | 14 | 4 | 4 |
| P18_2 | before | C | 58 | 13 | 2 | 2 |
| P2_1 | before | T | 14 | 7 | 4 | 2 |
| P2_2 | before | C | 20 | 9 | 0 | 0 |
| P20_1 | before | T | 5 | 5 | 2 | 2 |
| P20_2 | before | C | 21 | 16 | 2 | 2 |
| P5_1 | before | T | 28 | 14 | 3 | 2 |
| P5_2 | before | C | 9 | 5 | 1 | 1 |
| P6_1 | before | T | 44 | 21 | 10 | 5 |
| P6_2 | before | C | 23 | 14 | 7 | 4 |
| P7_1 | before | T | 50 | 14 | 4 | 2 |
| P7_2 | before | C | 26 | 11 | 0 | 0 |
| P8_1 | before | T | 11 | 7 | 6 | 3 |
| P8_2 | before | C | 93 | 36 | 3 | 2 |
| P9_1 | before | T | 9 | 6 | 0 | 0 |
| P9_2 | before | C | 9 | 6 | 4 | 2 |
