# Supplementary material for: Alkaline water as a potential agent for biting midge control: Managing effectiveness and non-target organism impact evaluation
Source: PLoS One. 2023 Aug 17;18(8):e0290262. doi: 10.1371/journal.pone.0290262 (PMC10434854; doi:10.1371/journal.pone.0290262)
Supplement: Attachment — Submitted filename: Response to reviewers_0531.docx [file pone.0290262.s002.docx]

Dear Dr. Samuel Adelani Babarinde

Thank you very much for granting us an opportunity to revise our manuscript PONE-D-23-06123 entitled “Alkaline water as a potential agent for biting midge control: Managing effectiveness and non-target organism impact evaluation“, and thank you and two reviewers for the constructive and insightful suggestions on how to improve our manuscript. We have revised the manuscript thoroughly following the comments and suggestions. Our point-by-point responses are provided below each comment, and our changes are tracked in the resubmitted manuscript, with line numbers specified in this reply letter.

In addition, we have carefully checked formatting, reference citations, reference list and spellings according to the latest PLOS ONE house style.

Best wishes,

I-Min Tso

------------------------------------------------------------------------------------------------------

**Responses to Journal Requirements:**

1. *Please ensure that your manuscript meets PLOS ONE's style requirements, including those for file naming.*

**Reply:** In this draft we have carefully checked formatting, reference citations, reference list and spellings according to the latest PLOS ONE house style.

2. *In your Methods section, please provide additional information regarding the permits you obtained for the work. Please ensure you have included the full name of the authority that approved the field site access and, if no permits were required, a brief statement explaining why.*

**Reply:** In our study the field experiment was conducted in the campus of our institute, Tunghai University, under the logistic support of Center for Environmental Protection and Occupational Safety and Health, Tunghai University and no permit was needed. In addition, in Taiwan no special permit is needed to study biting midges and commonly-seen invertebrates in the field.

a. *Please clarify the sources of funding (financial or material support) for your study. List the grants or organizations that supported your study, including funding received from your institution.*

**Reply:** the financial supports received by this study were as follows

National Science and Technology Council, Taiwan Grant (MOST 109-2311-B-029-001- MY3)

The Directorate General of Higher Education, Ministry of Education of the Republic of Indonesia

b. *State what role the funders took in the study. If the funders had no role in your study, please state: “The funders had no role in study design, data collection and analysis, decision to publish, or preparation of the manuscript.”*

**Reply:** The funders had no role in study design, data collection and analyses, decision to publish, or preparation of the manuscript.

c. *If any authors received a salary from any of your funders, please state which authors and which funders.*

**Reply:** The author Siti Latifatus Siriyah received a scholarship (partly) from the National Science and Technology Council, Taiwan Grant (MOST 109-2311-B-029-001- MY3), and also received a scholarship from The Directorate General of Higher Education, Ministry of Education of the Republic of Indonesia

4. *Please note that funding information should not appear in the Acknowledgments section or other areas of your manuscript. We will only publish funding information present in the Funding Statement section of the online submission form. Please remove any funding-related text from the manuscript and let us know how you would like to update your Funding Statement*.

**Reply:** We had removed the funding-related text from the Acknowledgement section of this draft of manuscript. (Lines 421-426).

5. *In your Data Availability statement, you have not specified where the minimal data set underlying the results described in your manuscript can be found….. All PLOS journals require that the minimal data set be made fully available…. Upon re-submitting your revised manuscript, please upload your study’s minimal underlying data set as either Supporting Information files or to a stable, public repository and include the relevant URLs, DOIs, or accession numbers within your revised cover letter.*

**Reply:** We had provided the minimal underlying data set of our study in a Supporting Information file.

------------------------------------------------------------------------------------------------------

**Responses to reviewers' comments:**

**Reviewer 1:**

1. *As this valuable study has been conducted in a local set, the authors should be more careful to avoid generalizing the results. We know that the further studies in different sets and conditions (influenced by climatically and soil characteristics), will guide us for further conclusions. The current study could be as one of the first valuable steps which should be followed by more detailed and different studies.*

**Reply:** In this study, we showed for the first time that spraying alkaline water was a feasible way to control biting midge in the field, at least in school campuses. We agree with the reviewer that our study was conducted in a local scale and it is not appropriate to use the study result generated from a single site to claim that such approach is universally effective. Therefore, in this draft we revised our conclusion according to the opinion of the reviewer by stating that despite the effectiveness of such approach in this present study, further studies are recommended to assess the potential influences of various environmental factors such as habitat types, elevation, temperature, soil composition, precipitation …etc. to further confirm the feasibility of this strategy in different areas. (Lines 414-418).

2. *Notable parts of introduction could and should be easily omitted (e.g., L64-68, 86-100). These and other parts could be summarized and only the parts which are needed and are in line with the results, should be emphasized.*

**Reply:** We followed the suggestion of the reviewer and relevant texts in the introduction section were deleted or condensed. (Lines 54-56)

3. *Also discussion could be revised. Some parts could be removed or moved to introduction (e.g., L340-349). Some parts of discussion are the repetition of the goals of study (e.g., L380-386).*

**Reply:** We followed the suggestion of the reviewer and relevant sentences were deleted from the introduction section.

**Reviewer 2:**

1. *It is a good effort made by the researchers, but it cannot be applied in the field. It is impossible to use its alkaline water in the field, so it will not practically solve a problem in public health.*

**Reply:** In this study, we provided empirical evidence from both laboratory and field experiments that spraying alkaline water could effectively reduce the density of biting midges without affecting non-target invertebrates important for ecosystem functioning. We consider spraying alkaline water a very feasible and applicable method form controlling biting midges in the field for the following reasons:

a. Our study sites were located in various parts of the campus of Tunghai University, Taichung City, Taiwan. The overall area of Tunghai University is about 140 ha and it has a diverse array of habitat types. We conducted manipulated studies in sites located in such heterogenous landscapes and found such method to be effective. Therefore, scientifically spraying alkaline water should be a feasible method in controlling biting midges.
b. Results of our study showed that a daily application of 20 liters of alkaline water per 100 square meters was effective in reducing microalgae abundance and biting midge density. We consider that such protocol should be easily applicable to urban localities suffering high densities of biting midges. Urban localities exhibiting high biting midge densities are those with high human population density and green spaces. It is relatively easy to generate enough amount of alkaline water by using industrial-scale ionic water generators to perform the spraying in such localities. For example, by using a regular industrial-scale ionic water generator a total of 360 liters of alkaline water could be produced in a night, which could be used to spray 1800 square meters of green spaces. Therefore, as was fully demonstrated in our study, with appropriate logistic supports such as water tanks, transportation vehicles, powered sprayers…etc. it is possible to use alkaline water to control biting midges in outdoor areas. Therefore, for urban localities exhibiting patches of green spaces situated between buildings and pavements such as elementary/middle school campuses and small to intermediate sized urban parks alkaline water is a potentially effective and eco-friendly way to control biting midges. We had supplemented a paragraph in the discussion section to describe the feasibility of such strategy in controlling biting midges in urban areas. (Lines 405-418)
