# Supplementary material for: Alkaline water as a potential agent for biting midge control: Managing effectiveness and non-target organism impact evaluation
Source: PLoS One. 2023 Aug 17;18(8):e0290262. doi: 10.1371/journal.pone.0290262 (PMC10434854; doi:10.1371/journal.pone.0290262)
Supplement: Attachment — Submitted filename: Response to reviewer 3rd draft_20230701.docx [file pone.0290262.s003.docx]

Dear Dr. Samuel Adelani Babarinde

Thank you very much for granting us an opportunity to revise our manuscript PONE-D-23-06123 entitled “Alkaline water as a potential agent for biting midge control: Managing effectiveness and non-target organism impact evaluation“, and thank you and two reviewers for the constructive and insightful suggestions on how to improve our manuscript. We have revised the manuscript thoroughly following the comments and suggestions. Our point-by-point responses are provided below each comment, and our changes are tracked in the resubmitted manuscript, with line numbers specified in this reply letter.

In addition, we have carefully checked formatting, reference citations, reference list and spellings according to the latest PLOS ONE house style.

Best wishes,

I-Min Tso

------------------------------------------------------------------------------------------------------

**Response to Reviewer**

**Reviewer #4**

1. *The manuscript is well written and revised. I might ask about the type of ions making water alkaline?*

**Reply:** As we mentioned in the method, the alkaline water used in our field experiments was produced by an ionic water generator, and there is no need to supplement additional chemical compound to the water.

2. *As the authors mentioned that this water (pH 10) had no effects on earth worm life and female crickets to oviposit. They examined the eggs weekly but no result about hatchability. Such females might be insensitive to the pH so they continued to lay eggs. So if embryos suffered mal-formation or decreased hatchability so this water would affect consequently ecosystem. Could the authors mention a sentence about hatchability of cricket’s eggs (%)?*

**Reply:** The concern of the reviewer is reasonable because if the egg hatching of crickets is affected then the roles crickets played in ecosystems will be affected. However, because of the following reasons we suggest that the egg hatchability of crickets should not be affected by the alkaline water used in this study. Firstly, crickets usually deposit the eggs deep in the soil rather than on the soil surface. When laying the eggs, female crickets will dig the soil using their front legs (ca <5 mm deep), then the females would insert the ovipositor and half of the abdomen in the hole they dig to release eggs (Réale & Roff, 2002). Therefore, the alkaline water sprayed on the soil surface should not affect the eggs too much. On the other hand, the effects of various pH on development and hatchability of cricket eggs are poorly understood. Most relevant studies focused on the effects of temperature and drought on cricket egg development. However, the study conducted in New Zealand showed that population density of *Hemiandrus* sp. (weta or giant flightless cricket) was not affected by soil properties including pH (Nboyine et al., 2016). Such result suggested that cricket egg hatchability and development should not be affected by the alkaline water we used in this present study. In this draft, we follow the suggestion of the reviewer and supplement several sentences to mention about the potential effects of alkaline water on egg hatchability of crickets (lines 370 - 381).

**Reference**

Nboyine JA, Boyer S, Saville D, Smith MJ, Wratten SD. 2016. Ground wētā in vines of the Awatere Valley, Marlborough: biology, density and distribution. *New Zealand Journal of Zoology*. 43: 336–350.doi:10.1080/03014223.2016.1193548.

Réale, D., & Roff, D. A. (2002). Quantitative genetics of oviposition behaviour and interactions among oviposition traits in the sand cricket. *Animal Behaviour*, 64(3), 397–406. https://doi.org/10.1006/anbe.2002.3084
